# Electrocardiographic Abnormalities and Mortality in Epilepsy Patients

**DOI:** 10.3390/medicina57050504

**Published:** 2021-05-16

**Authors:** Normunds Suna, Inga Suna, Evija Gutmane, Linda Kande, Guntis Karelis, Ludmila Viksna, Valdis Folkmanis

**Affiliations:** 1 Department of Infectology, Riga Stradins University, 3 Linezera St., LV-1006 Riga, Latvia; guntis.karelis@rsu.lv (G.K.); Ludmila.Viksna@rsu.lv (L.V.); 2 Department of Neurology and Neurosurgery, Riga East Clinical University Hospital “Gailezers”, 2 Hipokrata St., LV-1039 Riga, Latvia; suuna.inga@gmail.com; 3 Faculty of Medicine, University of Latvia, 19 Raina Blvd., LV-1586 Riga, Latvia; valdis.folkmanis@lu.lv; 4 Faculty of Residency, Riga Stradins University, 16 Dzirciema St., LV-1007 Riga, Latvia; evija.bumane@inbox.lv (E.G.); linda.kandee@gmail.com (L.K.)

**Keywords:** sudden cardiac death risk markers, epilepsy, short QT, electrocardiographic parameters, left ventricular hypertrophy, death in epilepsy

## Abstract

*Background and Objectives*: People with epilepsy (PWE) have a 2–3 times higher mortality rate than the general population. Sudden unexpected death in epilepsy (SUDEP) comprises a significant proportion of premature deaths, whereas sudden cardiac death (SCD) is among the leading causes of sudden death in the general population. Cardiac pathologies are significantly more prevalent in PWE. Whether electrocardiographic (ECG) parameters are associated with remote death in PWE has yet to be elucidated. The study objective was to assess whether interictal ECG parameters are associated with mortality in the long-term. *Materials and Methods*: The study involved 471 epilepsy patients who were hospitalized after a bilateral tonic-clonic seizure(s). ECG parameters were obtained on the day of hospitalization (heart rate, PQ interval, QRS complex, QT interval, heart rate corrected QT interval (QTc), ST segment and T wave changes), as well as reported ECG abnormalities. Mortality data were obtained from the Latvian National Cause-of-Death database 3–11, mean 7.0 years after hospitalization. The association between the ECG parameters and the long-term clinical outcome were examined. *Results*: At the time of assessment, 75.4% of patients were alive and 24.6% were deceased. Short QTc interval (odds ratio (OR) 4.780; 95% confidence interval (CI) 1.668–13.698; *p* = 0.004) was associated with a remote death. After the exclusion of known comorbidities with high mortality rates, short QTc (OR 4.631) and ECG signs of left ventricular hypertrophy (OR 5.009) were associated with a remote death. *Conclusions:* The association between routine 12-lead rest ECG parameters—short QTc interval and a pattern of left ventricular hypertrophy—and remote death in epilepsy patients was found. To the best of our knowledge, this is the first study to associate rest ECG parameters with remote death in an epileptic population.

## 1. Introduction

People with epilepsy have a mortality rate 2–3 times higher than that of the general population [1,2]. Causes of death in people with epilepsy can be classified into three groups: deaths that are unrelated to epilepsy, those that occur as a result of the underlying cause of epilepsy, and those in which the epilepsy itself is the cause of death [2,3]. Deaths occurring in status epilepticus and in accidents caused by seizures, such as drowning and burns, as well as those caused by aspiration, asphyxiation, and SUDEP (sudden unexpected death in epilepsy), are recognized as epilepsy-related deaths [3,4].

SUDEP is defined as a sudden, unexpected, witnessed or unwitnessed, non-traumatic, and non-drowning death in patients with epilepsy, with or without evidence of seizure, excluding documented SE, in which post-mortem examination does not reveal a toxicological or anatomical cause of death [5]. Cases where death is prevented by resuscitation are termed near-SUDEP. The standardized mortality ratio for SUDEP is nearly 24 times higher compared with sudden death in the general population [6]. SUDEP comprises between 2 and 18 per cent of all deaths in epilepsy [6,7]. When witnessed, most SUDEP cases occur following a convulsive seizure that triggers a severe alteration of respiratory and cardiac function leading to death [8,9], while SUDEP cases in the absence of preceding seizures have also been reported [10]. Current evidence suggests seizure-induced interrelated mechanisms involved in the development of SUDEP, including primary cerebral shutdown, cardiac arrhythmia, and central or obstructive apnea [11,12,13]. Centrally mediated hypoventilation has the predominant role in most recorded cases, whereas seizure-induced ventricular arrhythmia is a rare cause of SUDEP [14]. Cardiorespiratory findings are consistent with the mechanisms that were suspected to result in SUDEP more than two decades ago [15].

Cardiovascular disease is the leading cause of death worldwide [16]. SCD (sudden cardiac death) is among the leading causes of sudden death in the general population, especially in young men, accounting for 12–18% of total mortality [17,18,19]. While cardiovascular death rates have declined markedly over the past several decades, thus reducing the absolute rate of SCD, the incidence of SCD as a proportion of overall cardiovascular deaths has increased [20,21]. Community-based study data show that epilepsy is associated with a threefold increase in risk for sudden cardiac arrest (SCA), irrespective of the traditional cardiac risk factors for SCA. It is worth noting that 92% of the cohort population had no preceding seizure activity before cardiac arrest [22]. The prevalence of ECG (electrocardiographic) markers for SCA is increased in patients with refractory epilepsy [23].

Cardiac pathologies are significantly more prevalent in the epileptic population, with prevalence ratios of 1.8–2.5 [24,25,26]. A variety of cardiac and vascular conditions are more common in epilepsy patients, namely stroke, left ventricular hypertrophy, myocardial infarction, cardiovascular disease, peripheral artery disease, and diabetes [24,27,28]. The increased prevalence of cardiac health conditions in epilepsy is explained by different mechanisms of comorbidity: (a) comorbid cardiac or vascular disease causes epilepsy (e.g., remote stroke causes epilepsy), (b) epilepsy and its treatment cause comorbid conditions, and (c) shared underlying mechanisms cause both conditions [29].

There are multiple mechanisms by which the increased risk for cardiac abnormalities in epilepsy patients can be explained. (1) Cardiac autonomic function may be impaired in epilepsy patients as evidenced by myocardial SPECT [30] and heart rate variability (HRV) studies [31,32], with research results showing that cardiac autonomic dysfunction exists even in patients with newly diagnosed untreated epilepsy [33]. HRV progressively worsens if seizures are refractory to treatment [34].

(2) In addition to structural changes caused by comorbid cardiac disease, microstructural changes in the heart are also reported (e.g., perivascular and interstitial myocardial fibrosis, myofibrillar degeneration), for which recurrent seizures are believed to be the cause [35,36,37]. Seizure-related mechanisms of myocardial injury are attributed to ictal hypoxemia [38], myocardial ischemia, the cardiotoxic effects of excess catecholamines, and coronary vasospasm, resulting in myocardial cell loss and fibrosis [36,37].

(3) Ictal hypoxemia causes transient cardiac repolarization abnormalities with either a lengthening or shortening of the QTc interval (heart rate corrected QT interval) [39,40,41]; permanent repolarization abnormalities also exist. Altered interictal cardiac repolarization (e.g., prolongation [42,43] or shortening [44] of the QT interval) is found in people with epilepsy. While mean QTc intervals lie within normal ranges in most studies [44,45], an increased prevalence of abnormal QTc prolongation has been reported [23,46].

(4) The cardiac ion-channels that are responsible for normal repolarization may be dysfunctional in epilepsy patients [47]. Defects in ion channel function can be caused by either genetic or acquired factors [48]. The pathophysiology of an acquired cardiac channelopathy in epilepsy is not fully understood; possible mechanisms include the overstimulation of autonomic pathways, which may mediate the transcriptional dysregulation of cardiac ion channels, as well as structural cardiac abnormalities, causing ion channel remodeling [49]. Genes which encode ion channels are expressed in the brain and heart tissue. There is evidence from animal and human studies that the same gene mutations may predispose for both epilepsy and cardiac arrhythmia [50,51]. Multiple gene mutations linked to epilepsy and arrhythmia in humans have also been discovered, such as the ones in voltage-gated potassium channel α-subunit encoding gene *KCNH2* [52,53,54], voltage-gated sodium channel α-subunit encoding gene *SCN5A* [55,56], and cardiac ryanodine receptor encoding gene *RYR2* [57].

The concept of “the epileptic heart” has recently been postulated, and is defined as “a heart and coronary vasculature damaged by chronic epilepsy as a result of repeated surges in catecholamines and hypoxemia leading to electrical and mechanical dysfunction” [58].

In the context of the current understanding of cardiac disease in epilepsy patients, which, in addition to the classical cardiac risk factors, is affected by recurring pathologic central nervous system (CNS) effects in the form of seizures, the current study addresses ECG data and markers of SCD in particular, as well as data of long-term mortality in these patients.

## 2. Materials and Methods

### 2.1. Study Population

A retrospective cross-sectional study was conducted in the Neurology Department of Riga East Clinical University Hospital Gailezers. Data were collected from medical records on all epilepsy patients (*n* = 544) who were admitted to hospital after an epileptic seizure between January 2006 and December 2014. The inclusion criteria were as follows: patients 18 years of age and older, one or more bilateral tonic-clonic seizure(s) before admission to hospital, a discharge diagnosis of epilepsy (previously known or newly diagnosed epilepsy), and the data necessary for the study being available in the medical records. Exclusion criteria were as follows: medical records containing incomplete data, oncological disease in the anamnesis or current oncological disease, the patient having been admitted to hospital with a different type of seizure that was not a bilateral tonic-clonic seizure; and acute symptomatic seizure.

### 2.2. Materials

According to the inclusion and exclusion criteria, the data from 471 out of 544 patients were further analyzed in the study. The main parameters collected and used in the data analysis were as follows: cause of epilepsy, frequency of seizures, age, gender, arterial blood pressure measurement from the Clinic of Emergency Medicine and Patient Admission, and ECG data obtained on the first day of hospitalization (heart rate, PQ interval, QRS complex, QT/QTc interval, ST segment and T wave changes), as well as ECG abnormalities reported by cardiologist, and the long-term outcome of the epilepsy. Epilepsy was diagnosed by a certified neurologist, and the ECG report was provided by a certified cardiologist. In addition, the fact of death was confirmed from the Latvian National Cause-of-Death database on 16 August 2017. The cause of death was not analyzed in the study to avoid possible inaccuracies in the death certificates.

The study analyzed the ECGs that were recorded within 24 h of the patient’s admission to hospital. The heart rate corrected QT (QTc) interval was automatically determined during the recording of a 12-lead rest ECG with an electrocardiograph. If the QTc was not determined automatically, it was calculated manually using the Framingham formula (QTc = non-corrected QT + 154 × (1-heart rate)) [59], since it matched the method used in the electrocardiograph. QTc measurements ≥450 ms in men and ≥460 ms in women were considered abnormally prolonged, the QTc intervals <360 ms were considered to be short [60]. The second QTc correction method, the Fridericia formula, was used for the verification of the study results [61]. All ECGs were evaluated and the clinical interpretations were provided by a certified cardiologist, data were also compiled from the cardiologist’s conclusion regarding the ECG pathology (nonspecific ST segment and T wave changes, nonspecific changes in ECG, intraventricular and intra-atrial conduction disturbances, signs of left ventricular hypertrophy, early ventricular repolarization pattern (ERP), atrioventricular block, pathologic Q wave changes, Wolf–Parkinson–White syndrome (WPW), atrial fibrillation).

The study was conducted in full compliance with the applicable legal and regulatory requirements. Ethical approval for this study was obtained from the Medical and Biomedical Research Ethics Committee of the Riga East Clinical University hospital (4 September 2014, Reference No. 12-A/14/04.09.2014).

### 2.3. Statistical Analyses

Patients were divided into two groups for data analysis: (1) patients whose fact of death was not registered in the Latvian National Cause-of-Death database and (2) patients who were registered in the Latvian National Cause-of-Death database at the time of evaluation (16 August 2017).

IBM SPSS (version 22.0) software (IBM Corporation, New York, NY, USA) was used for the statistical processing and analysis of the data. Descriptive statistics for categorical variables—frequency (in absolute numbers and as a percentage), and the central tendency for quantitative variables—the mean value and standard deviations (for normally distributed data), or median values and interquartile range (for non-normally distributed data) were used. A Shapiro–Wilk test was used to assess the normal distribution; in the case of a normal distribution the relationship between the different nominal dependent and independent variables were assessed using either a T-test or an ANOVA test, depending on the number of lines, but in the case of categorical variables, a chi-square test was used. The logistic regression (univariate and multivariate) method was used for further data analysis to examine the associations between the clinical outcome (patient data in the Cause-of-Death database) and the ECG parameters, adjusting the data according to age and gender.

## 3. Results

### 3.1. Demographic and Clinical Characteristics

Of all the epilepsy patients (*n* = 471) included in the study, women comprised 45.9% (*n* = 216), men—54.1% (*n* = 255). The average patient age was 47.2 (standard deviation (SD) 18.75), most were young working-age people. Mortality data was available for 467 patients, or 99.2% of the study population, of whom 75.4% (*n* = 352/467) were alive at the time of analysis, but 24.6% (*n* = 115/467) were deceased. The mean age in the alive patient group was 43.2 (SD 17.1), while in the deceased patient group it was 59.2 (SD 18.3) years (*p* < 0.005). At the time of ascertaining the cause of death, 3–11 years (median 7.0) had passed since being discharged from hospital. Of all patients included in the study, 22.2% (*n* = 48/216) of the women had died and 26.7% (*n* = 67/251) of the men had died, *p* = 0.283.

Of the illnesses that can directly affect ECG due to myocardial changes, the following comorbidities were found in the study population: arterial hypertension in 32.1% (*n* = 151), congestive heart failure—10.2% (*n* = 48), coronary heart disease—11.7% (*n* = 55), old myocardial infarction—6.4% (*n* = 30), unspecified cardiomyopathy—4.9% (*n* = 23), atrial fibrillation (permanent or paroxysmal form)—4.9% (*n* = 23), and congenital heart defects in 0.2% (*n* = 1) of patients.

### 3.2. Epilepsy Characteristics

Only epilepsy patients who were admitted to hospital after a bilateral tonic-clonic seizure(s) were analyzed (*n* = 471). Of all patients who had a discharge diagnosis of epilepsy, a newly diagnosed epilepsy was present in 16.8% (*n* = 79/471) of patients and known epilepsy in 82.4% (*n* = 388/471); however, the previous status of epilepsy diagnosis was unknown in 0.8% (*n* = 4/471) of patients. Focal epilepsy was diagnosed in 76.4% (*n* = 360/471), generalized epilepsy—11.0% (*n* = 52/471), and epilepsy of an unknown type in 12.5% (*n* = 59/471) of the study population [62].

Data on the average frequency of seizures were available in the medical records of 54.4% (*n* = 211/388) of patients with previously known epilepsy, and, accordingly, 6.2% (*n* = 32/211) of patients had seizures every day, 16.6% (*n* = 44/211) every week, 29.9% (*n* = 63/211) every month, 26.5% (*n* = 56/211) less than once a month, and 20.9% (*n* = 44/211) of patients had seizures one or fewer times a year.

Of the total study population, 41.4% (*n* = 195) of the patients had a recurrent seizure(s) within 24 h before admission to hospital. 24.8% of all patients included in the study had a recurrent seizure(s) during the hospitalization period, where 16.6% (*n* = 78/471) had one within the first 24 h; 13.2% (*n* = 62/471) after more than 24 h, and 4.9% (*n* = 23/471) had recurrent seizures both within and after the first 24 h period. Seizure clusters were documented in 17.4% (*n* = 82/471) of patients, of whom 14.6% (*n* = 12/82) were admitted to the intensive care unit (ICU). Status epilepticus was observed in 6.8% (*n* = 32/471) of patients, of whom 71.9% (*n* = 23/32) were admitted to the ICU. A total of 7.4% (*n* = 35/471) of the patients included in the study required treatment in the ICU.

### 3.3. ECG Parameter Characteristics

Data on the 12-lead rest ECG record within 24 h of being admitted to hospital were available for 93.8% (*n* = 442/471) of the patients included in the study; ECG data were not available for 6.2% (*n* = 29/471). Of all patients with ECG reports available, five did not have data on the QTc interval (*n* = 5/442). Mean values of available ECG parameters were as follows: heart rate of 81.2 (SD 19.7) beats per minute; PQ interval—159.4 (SD 23.9) ms, and QRS interval—93.8 (SD 15.8) ms. Of the patients with available data on the QTc interval (*n* = 433/471), 4.4% (*n* = 19/433) of patients were determined to have a short QT interval; however, 5.3% (*n* = 23/433) of patients had a long QT interval. The mean QTc was 402.5 (SD 27.7), the mean QTc for alive patients was 402.3 ms (SD 26.2), and the mean QTc for the deceased was 402.7 ms (SD 31.4) (*p* = 0.524). The mean QT interval for women was 374.8 (SD 38.06) ms and the QT interval for men was 364.65 (SD 39.8) ms, while the mean QTc interval for women was 406.2 (SD 26.4) ms and the QTc interval for men was 399.3 (SD 28.4) ms.

Out of all the recorded ECGs, 26.0% (*n* = 115/442) of patients were determined to have nonspecific ST segment and T wave changes and 11.8% (*n* = 52/442) of patients were found to have nonspecific ECG changes. Intraventricular conduction disturbances were observed in 20.6% (*n* = 52/442), intra-atrial conduction disturbances in 6.6% (*n* = 29/442), signs of left ventricular hypertrophy in 12.2% (*n* = 54/442), signs of ERP in 1.6% (*n* = 7/442), atrioventricular block (of any degree) in 2.9% (*n* = 13/442), pathologic Q wave changes in 5.4% (*n* = 24/442), and WPW (Wolf–Parkinson–White syndrome) pattern in 0.5% (*n* = 2/442) of the patients.

### 3.4. ECG Parameters in Univariate Analysis

Univariate logistic regression analysis was conducted on the patients who had available ECG (*n* = 442) and mortality data (*n* = 438). Of the patients whose ECG and mortality data were available (*n* = 438), 75.3% (*n* = 330) were alive, but 24.7% (*n* = 108) were deceased. Of the 438 patients, five did not have available QTc interval data, therefore this parameter was analyzed in a group of 433 patients.

Of the risk markers of SCD found in the general population, the study population were registered to have the following ECG parameters: short QTc interval (3.4% in the alive patients group vs. 7.5% in the deceased patients group, *p* = 0.068), long QTc interval (4% vs. 9.4%, *p* = 0.043), ERP (1.8% vs. 0.9%, *p* > 0.999), and WPW (0.3% vs. 0.9%, *p* = 0.433).

The aforementioned groups had the following other ECG parameters: nonspecific ST segment and T wave changes (22.7% in the alive patient group vs. 35.2% in the deceased patient group, *p* = 0.016), intraventricular conduction disturbances (19.1% vs. 24.1%, *p* = 0.0272), intra-atrial conduction disturbances (4.2% vs. 13.9%, *p* = 0.001), left ventricular hypertrophy (8.2% vs. 25%, *p* < 0.005), pathologic Q wave changes (3.3% vs. 11.1%, *p* = 0.004), and atrioventricular block (2.4% vs. 4.6%, *p* = 0.323) (Table 1).

Determinants that were univariately associated with the fact of being deceased (*p* values < 0.05) were included in the multivariate analysis, in addition to the risk markers of SCD described in the literature. Overall, the following determinants were used in further analysis: short QTc, long QTc, nonspecific ST segment and T wave changes, intra-atrial conduction disturbances, left ventricular hypertrophy, pathologic Q wave changes, ERP, and WPW syndrome.

### 3.5. ECG Parameters in Multivariate Analysis

In the multivariate logistic regression analysis, death in the late period after hospital discharge had no statistically significant association with the following determinants: ERP (OR 0.942, CI (0.103–8.586), *p* = 0.958),WPW (OR 2.107; CI (0.042–105.865), *p* = 0.708), long QTc interval (OR 1.259, CI (0.479–3.314), *p* = 0.640), long QTc interval (Fridericia) OR 1.831, CI (0.666–5.032, *p* = 0.241), nonspecific ST segment and T wave changes (OR 0.792, CI (0.460–1.363), *p* = 0.399), pathologic Q wave changes (OR 1.673, CI (0.640–4.371), *p* = 0.294), or intra-atrial conduction disturbances (OR 2.231; CI (0.952–5.228), *p* = 0.065), adjusting data for age and gender.

It was shown by multivariate analysis that remote death after hospital discharge had a significant association with the following determinants: age (OR 1.047; CI (1.031–1.064), *p* < 0.005), short QTc interval (OR 4.780, CI (1.668–13.698), *p* = 0.004), short QTc interval (Fridericia) OR 3.153, CI (1.308–7.602, *p* = 0.011), and left ventricular hypertrophy (OR 0.458; CI (0.235–0.893), *p* = 0.022), adjusting data for age and gender.

### 3.6. Ruling Out Potentially Fatal Known Comorbidities

To rule out the effect of cardiac comorbidities on ECG parameters and to decrease the effects of other serious health conditions on long-term mortality, patients (*n* = 150/471) with the known following comorbidities were excluded from further data analysis: alcohol and drug dependence, HIV infection, viral hepatitis, chronic kidney disease, glomerulonephritis, chronic obstructive pulmonary disease, bronchial asthma, cerebrovascular disease, previous stroke and intracerebral hemorrhage, diabetes mellitus, congenital heart defects, cardiomyopathy, fibrillation, coronary heart disease, acute coronary syndrome, and previous myocardial infarction.

In this subpopulation with the aforementioned excluded comorbidities (*n* = 321), the mean age of patients was 40.9 (SD 16.20). Women constituted 46.4% (*n* = 149/321), and men—53.6% (*n* = 172/321). Data on the fact of death were not available for two patients (*n* = 2/321). Of the subpopulation, 17.2% (*n* = 55/319) were deceased, of whom 23 were women and 32 were men, but 82.8% (*n* = 264/319) of the patients were alive.

An ECG had been recorded in 91.9% (295/321) of the patients in this subpopulation. QT interval data were available for 98.6% (*n* = 291/295) of the patients; mean QTc 399.82 ms (SD 26.6). In the deceased group, mean QTc was 393.77 (SD 31.2), and in the alive group, mean QTc was 400.99 (SD 25.5), *p* = 0.29. Data on nonspecific ST segment and T wave changes were available for 99.3% (*n* = 293/295) of patients.

In the patients whose ECG (*n* = 295) and mortality data (*n* = 293) were available, 83.6% (*n* = 245/293) were alive and 16.4% (*n* = 48/293) were deceased. Univariate analysis data of the patient subpopulation (*n* = 293) is presented in a table form (Table 2). A statistically significant difference between the groups (alive vs. deceased) was discovered for left ventricular hypertrophy (4.5% vs. 20.8%, *p* < 0.005) and short QTc interval (3.7% vs. 10.6%, *p* = 0.042).

Determinants that were univariately associated with the fact of being deceased (*p* values < 0.05) were included in the multivariate logistic regression analysis, in addition to the risk markers of SCD described in literature: long QTc, short QTc, non-specific ST segment and T wave changes, intra-atrial conduction disturbances, left ventricular hypertrophy, pathologic Q wave changes, ERP, and WPW syndrome.

In the multivariate analysis, remote death after hospital discharge had no statistically significant association with the following parameters: ERP (OR 1.206, CI (0.127–11.433), *p* = 0.870), WPW (OR 0; CI (0.00–0), *p* > 0.999), long QTc interval (OR 1.280, CI (0.251–6.515), *p* = 0.766), long QTc interval (Fridericia) OR 3.044, CI (0.523–17.723, *p* = 0.213), intra-atrial conduction disturbances (OR 1.568, CI (0.427–5.755), *p* = 0.498), pathologic Q wave changes (OR 0.762; CI (0.088–6.631), *p* = 0.806), or nonspecific ST-T changes (OR 1.712; CI (0.787–3.723), *p* = 0.175), adjusting data for age and gender.

It was shown in the multivariate analysis that remote death after hospital discharge had a significant association with the following determinants: short QTc interval (OR 4.631, CI (1.378–15.560), *p* = 0.013), short QTc interval (Fridericia) OR 3.123, CI (1.023–9.532, *p* = 0.046), left ventricular hypertrophy (OR 5.009; CI (1.829–13.721), *p* = 0.002), and age (OR 1.033, CI (1.011–1.055), *p* = 0.003).

## 4. Discussion

The study included all patients who were admitted to hospital after bilateral tonic-clonic seizures between 2006 and 2014. Patients with focal onset seizures that did not progress to bilateral tonic-clonic seizures and patients with isolated absence or myoclonic seizures were not included because the precise quantification of such seizures is not possible and would create inaccuracies in describing the degree to which the epileptic seizures were controlled. Riga East Clinical University hospital is one of the two university hospitals in Latvia, and with a large proportion of the population living in or near the capital city, Riga, the catchment area for both hospitals includes roughly half of the country’s population [63]. Patients with different degrees of seizure control were represented in the study population, namely 16.8% of patients were with newly diagnosed epilepsy. Among the patients whose frequency of seizures was known, they occurred once a year or less for one fifth of patients, therefore the study did not represent only drug-resistant epilepsy cases. It is known that the highest standardized mortality ratios are seen during the first 5–10 years after diagnosis, and from then on remain stable or decline [64], thus verifying the registered fact of death in the Latvian National Cause-of-Death database 3–11 (median 7.0 years) years after being discharged from hospital made it possible to obtain a large group of patients who were not alive during the study, i.e., 24.6%. The proportion of epilepsy patients who were deceased is comparable with the data from other studies, for example, in a population-based study with a reference period of 6.9 years, 20.2% of patients with a definite epilepsy diagnosis had died [1].

Studies that are conducted on ECG parameters in epileptic populations usually look at general ECG variables (heart rate, rhythm, PR interval, duration of QRS, QTc interval) [42,46,65,66,67], and mostly conduct analysis on the ventricular repolarization phase (QT interval). QTc has been addressed in isolation in few studies [44,68]. Even though studies yield conflicting findings, the results that indicate longer QTc in epilepsy patients dominate [23,42,46,65,66,67]. Data from other studies show QTc that are either not different [66] or are shorter compared to control populations [44,67].

The number of studies that analyze the risk markers of cardiac death is limited. One study describes three markers of SCA risk, of which QTc prolongation (male > 450 ms, female > 470 ms) and early repolarization pattern were more prevalent in epilepsy patients, while Brugada ECG pattern was not [23]. Another study reports that early repolarization (ER) pattern and Brugada type ECG pattern (BP) were significantly more prevalent in subjects without epilepsy [46]. One study provides a detailed description of ECG abnormalities, including atrial enlargement, ventricular hypertrophy, bundle-branch blocks, fascicular blocks, AV block, and pathologic Q and T waves. A more prolonged P-wave and PR interval, longer QT intervals, pathologic QT dispersion, and left atrial overload were found in epilepsy patients compared to the control population in this study [69]. A compilation of interictal parameters from several ECG studies has been performed elsewhere [69,70].

Even though the ECG changes in epilepsy patients have been reported and the risk markers of SCD in the general population are also known, the association between these parameters and death in the epileptic population has not been elucidated. Previous studies have focused on cardiac marker association with the risk of SUDEP. Evidence of ECG abnormalities provided by a matched case–control study shows that SUDEP patients more frequently have an abnormal ventricular conduction ECG pattern (58% vs. 18%, *p* = 0.04), while early repolarization was similarly prevalent in cases and controls [71]. Another case–control study did not find an association of SUDEP with preictal QTc, ictal QTc, postictal QTc, QRS, heart rate, or HRV [43].

The current study compared general ECG variables and markers of SCD in epilepsy patients in populations of both alive and deceased individuals at the time of data examination in a late period after being discharged from hospital. Cause of death was not known, therefore the study results only allow for an overall assessment of the risk of death. When the effect of anti-epileptic treatment on mortality is addressed, it has to be stressed that the question cannot be answered by the current study when taking into account the period of many years following hospitalization until the evaluation of the cause of death. The treatment could have been changed over time due to drug-resistance or side effects. Anti-epileptic drugs do not seem to increase the risk of SCD. While increased risk for SCD has been reported by the results of a community-based study for carbamazepine and gabapentine [72], it was later criticized for possible indication bias affecting the results [73].

This study identified a pathologically prolonged interictal QTc in 5.3% (*n* = 23/433) of patients, which is similar to what is reported in other studies where 5% of epilepsy patients were determined to have a prolonged QTc [23]; in another study, prolonged QTc was found in 8.4% of patients, but borderline QTc prolongation with QTc interval thresholds of ≥440 in males and ≥450 in females was used [46]. In the current study, prolonged QTc was not associated with death at a late period after discharge, as shown by multivariate analysis either in the whole epileptic population (OR 1.259, CI (0.479–3.314), *p* = 0.640) or in the subpopulation with excluded serious comorbidities (OR 1.280, CI (0.251–6.515), *p* = 0.766). Even though the prevalence of prolonged QTc was greater in the patient group with significant comorbidities compared to the group without comorbidities, the difference was not statistically significant (*p* = 0.089). An explanation for this lack of association with increased mortality risk could be the magnitude of QTc prolongation, because the QTc of only two patients was 500 ms or more, only one could be classified as high-risk QTc prolongation (>500 ms) [74,75], and another three exceeded the upper limit of “borderline” QTc prolongation [76]. We used automatic QTc detection by an electrocardiograph, while manual QTc measurements, rather than automatic assessments, are advocated by Long QT syndrome experts, and the cutoff values for the different correction methods, age, and gender are provided [77].

A threshold of <360 ms for short QTc interval, a criterion used in the diagnostics of short QT syndrome rather than of shortened QTc in the general population, was chosen. Studies reporting shorter QTc in epilepsy patients showed that epilepsy patients have 15–20 ms shorter mean QTc intervals compared to control populations [67], and in epilepsies of unknown etiology (cryptogenic), the mean QTc overlaps the threshold used for defining shortened QTc in the general population (<390 ms), thus such a threshold would result in a substantial number of false positive results [44]. Short QTc was associated with remote mortality in the baseline epileptic population OR 4.780, CI (1.668–13.698, *p* = 0.004) and in the subpopulation with excluded potentially serious comorbidities OR 4.631, CI (1.378–15.560, *p* = 0.013).

In order to reaffirm the study results, the second correction factor, the Fridericia formula, was used. In the multivariate logistic regression analysis, death in the late period after hospital discharge had statistically significant association with short QTc interval (Fridericia) OR 3.153, CI (1.308–7.602, *p* = 0.011). The association remained statistically significant in the subpopulation with excluded comorbidities, as well: short QTc intervals (Fridericia) OR 3.123, CI (1.023–9.532, *p* = 0.046). Prolonged QTc was not significantly associated with remote death when either the Fridericia or Framingham correction method was used.

The frequency of early repolarization pattern did not differ between the alive and deceased patient groups (*p* > 0.999), it was found only in 1.6% of patients; it is also substantially less frequently than in other studies, where such a finding was reported in 9.7–34% of patients [23,69,78]. Abnormal early repolarization has recently been found to be more prevalent in epilepsy patients than in controls. It is not altered by antiepileptic drugs or by achieving the suppression of epilepsy [78].

No patients included in the study were found to have a Brugada ECG pattern, although the finding is rare in European populations—0–0.6% [79].

Nonspecific ST and T wave changes were the most common ECG abnormality, which was found in 26% of patients. The pathology was more frequent in deceased patients in the univariate analysis (*p* = 0.016), but the difference was negated when serious comorbidities were excluded, which is not surprising when taking into account the association of nonspecific ST and T abnormalities with such common comorbidities as hypertension, diabetes, and cardiovascular disease [80].

Signs of left ventricular hypertrophy had a positive association with remote death only after excluding known serious comorbidities OR 5.009, CI (1.829–13.721, *p* = 0.002).

Of the remaining parameters analyzed in the univariate analysis, the deceased patients were more often found to have intra-atrial conduction disturbances (*p* = 0.001), left atrial overload (*p* = 0.042), and pathologic Q wave changes (*p* = 0.004); however, the association of these parameters with remote death was not confirmed in the multivariate analysis.

Even though the cause of death was not analyzed in this study, it was most likely cardiac death. The exact mechanism of death in the epileptic population with the aforementioned ECG markers could be proven via a matched case–control study design in patients with a known cardiac cause of death.

## 5. Conclusions

This study found an association between routine 12-lead rest ECG parameters—short QTc interval and a pattern of left ventricular hypertrophy—and remote death in epilepsy patients. To the best of our knowledge, this is the first study to associate rest ECG parameters with remote death in an epileptic population.

## Figures and Tables

**Table 1:** Electrocardiographic parameters in the study population (*n* = 438).

Parameter	Alive and the Parameter Was Present	Deceased and the Parameter Was Present	*p* Value
Short QTc (*n* = 19)	3.4% (*n* = 11)	7.5% (*n* = 8)	0.068
Long QTc (*n* = 23)	4% (*n* = 13)	9.4% (*n* = 10)	0.043
Short QTc Fridericia (*n* = 29)	5.5% (*n* = 18)	10.4% (*n* = 11)	0.080
Long QTc Fridericia (*n* = 20)	3.3% (*n* = 11)	8.3% (*n* = 9)	0.067
Nonspecific ST-T changes (*n* = 113) *	22.7% (*n* = 75)	35.2% (*n* = 38)	0.016
Intraventricular conduction disturbances (*n* = 89) *	19.1% (*n* = 63)	24.1% (*n* = 26)	0.272
Intra-atrial conduction disturbances (*n* = 29)	4.2% (*n* = 14)	13.9% (*n* = 15)	0.001
Left ventricular hypertrophy (*n* = 54)	8.2% (*n* = 27)	25% (*n* = 27)	<0.005
Pathologic Q wave changes (*n* = 23) *	3.3% (*n* = 11)	11.1% (*n* = 12)	0.004
Atrioventricular block (*n* = 13)	2.4% (*n* = 8)	4.6% (*n* = 5)	0.323
Early repolarization (*n* = 7)	1.8% (*n* = 6)	0.9% (*n* = 1)	>0.999
WPW syndrome (*n* = 2)	0.3% (*n* = 1)	0.9% (*n* = 1)	0.433

*—total number of patients differs from the number in the described study population because the mortality data of 4 patients were not available. QTc: Heart rate corrected QT interval. WPW: Wolf–Parkinson–White syndrome.

**Table 2:** Electrocardiographic parameters in the subpopulation with excluded comorbidities (*n* = 293).

Parameter	Alive and the Parameter Was Present	Deceased and the Parameter Was Present	*p* Value
Short QTc (*n* = 14)	3.7% (*n* = 9)	10.6% (*n* = 5)	0.042
Long QTc (*n* = 10)	2.9% (*n* = 7)	6.4% (*n* = 3)	0.208
Short QTc Fridericia (*n* = 19)	5.7% (*n* = 14)	10.4% (*n* = 5)	0.226
Long QTc Fridericia (*n* = 7)	1.6% (*n* = 4)	6.3% (*n* = 3)	0.055
Nonspecific ST-T changes (*n* = 63)	20.0% (*n* = 49)	29.2% (*n* = 14)	0.157
Intraventricular conduction disturbances (*n* = 55)	18.4% (*n* = 45)	20.8% (*n* = 10)	0.689
Intra-atrial conduction disturbances (*n* = 15)	4.1% (*n* = 10)	10.4% (*n* = 5)	0.079
Left ventricular hypertrophy (*n* = 21)	4.5% (*n* = 11)	20.8% (*n* = 10)	<0.005
Pathologic Q wave changes (*n* = 5)	1.2% (*n* = 3)	4.2% (*n* = 2)	0.190
Atrioventricular block (*n* = 6)	2.4% (*n* = 6)	0% (*n* = 0)	0.594
Early repolarization (*n* = 7)	2.4% (*n* = 6)	2.1% (*n* = 1)	>0.999
WPW syndrome (*n* = 1)	0.4% (*n* = 1)	0% (*n* = 0)	>0.999

## Data Availability

The data presented in this study are available on request from the corresponding author. The data are not publicly available due to ethical restrictions.
